# Instruments for measuring incidents related to patient safety in the context of paediatric intensive care—protocol for a scoping review

**DOI:** 10.1186/s13643-022-01888-6

**Published:** 2022-01-25

**Authors:** Helga Catarina Santos Alves de Oliveira, Ricardo Rafael Marques, Maria Alice dos Santos Curado, Maria Filomena Mendes Gaspar, Paulo Jorge dos Santos Sousa

**Affiliations:** 1 Escola Superior de Enfermagem de Lisboa, Universidade de Lisboa, Av. D. João II, Lote 4.69.01, 1990-096 Lisbon, Portugal; 2 Centro de Investigação, Inovação e Desenvolvimento em Enfermagem de Lisboa, Lisbon, Portugal; 3 Universidade NOVA de Lisboa; Comprehensive Health Research Center, Universidade NOVA de Lisboa, Lisbon, Portugal

**Keywords:** Paediatric intensive care, Patient safety, Measurement tool, incident, Adverse event

## Abstract

**Background:**

Patient safety is a fundamental principle of health care but is one of the biggest challenges currently faced and a serious public health problem, since the occurrence of adverse events is probably one of the main causes of morbidity and mortality worldwide. The vulnerability of the paediatric population, combined with the potentially dangerous context of intensive care, makes Paediatric Intensive Care Units services of particular complexity in matters of safety, where there is a greater likelihood of incidents with serious consequences. It is agreed that research on the topic of PS should start with the measuring of different types of harm that exist in the contexts, to identify high-risk areas and define priorities. For this, it is necessary to resort to a multiplicity of valid, reliable and specific measurement instruments and to learn their advantages and limitations. The objective of this review will be to identify and map in scientific literature the instruments for measuring incidents related to patient safety applicable in the context of paediatric intensive care.

**Methods:**

This review will cover studies and documents that refer to all measurement instruments used in the field of patient safety in a context of paediatric intensive care. Quantitative, qualitative, or mixed nature published studies, as well as grey literature, produced in the last 5 years and relevant to the topic will be included, in Portuguese, English or Spanish languages. The sources of information include several databases (such as MEDLINE, CINAHL, Cochrane Library, JBI Databases) and sources relevant to grey literature. Two reviewers will independently screen all citations, full-text articles and abstract data. The extracted data, after being organised in the extraction table, will be mapped in a descriptive and logical way, taking into account the defined review questions.

**Discussion:**

The mapping of the tools in these protocols will allow to summarise the most widely used instruments, to know their specificities and to guide researchers to use the most appropriate measurement tools for their context, specifically, in paediatric intensive care.

**Systematic review registration:**

Open Science Framework (osf.io/b5m7j).

**Supplementary Information:**

The online version contains supplementary material available at 10.1186/s13643-022-01888-6.

## Background

### Health safety

From a historical point of view, “Primum non Nocere”, or “First, do no harm”, evoked by Hippocrates, was the first reference to the subject of patient safety [PS] [1]. Florence Nightingale, in an embryonic era for health care, also acknowledged that the conditions in which care is provided are defining [2, 3]. It was this pioneer who first approached the dimension of quality, but it is Avedis Donabedian who is attributed with the first concrete definition, composed of the triad result, process and structure. Later, the Institute of Medicine [IOM] integrated safety as an inseparable pillar of quality, and the dimension of satisfaction was also added, from the perspective of patient-centred care [1].

To better understand this topic, it is important to clarify some key concepts of taxonomy defined by the World Health Organization [WHO] and translated by the General Directorate of Health [4]:

- Patient Safety is reducing the risk of unnecessary harm, related to healthcare, to an acceptable minimum. Risk is the probability of an incident occurring;
- Quality is the degree to which health services increase the likelihood of desired health outcomes and are consistent with current professional knowledge;
- An incident can be a reportable occurrence, a quasi-event, and an incident without harm or an incident involving harm (adverse events [AE]). Harm implies damage to the structure or functions of the body and/or any resulting harmful effects, including injury, suffering, disability or death, and may be physical, social or psychological;
- Error is the failure to execute a planned action according to the desired or the incorrect development of a plan.

The subject of patient safety has, in recent decades, become a central issue on health and society agendas the world over. After the revolutionary To Err is Human report carried out in 2000 by the IOM [5], which reported a remarkable number of deaths due to errors in the health system, several safety agencies were created, including the Agency for Healthcare Research and Quality, the Institute for Healthcare Improvement [IHI] and the World Alliance for Patient Safety. Since then, several studies have been carried out and bolstered the data published by the IOM [6]. According to the WHO [7, 8], harm to the patient currently represents the 3rd leading cause of death in the United States of America [USA], and it is estimated that one in ten patients suffers an event involving harm while receiving hospital care, of which about 50% would be preventable. The economic costs associated with medication errors total around $42 billion, 15% of healthcare spending is derived from AE, and security strategies represented savings of $28 billion in the USA between 2010 and 2015. At European level, it is estimated that 8–12% of hospitalised patients are subject to the occurrence of an AE [9].

As can be seen, PS is currently a serious public health problem that needs an effective response. The strategies adopted must be adaptable to local contexts and available resources [10] and, among the various proposed government actions, the following stand out: the creation of specialist agencies, the implementation of a national incident reporting system, the promotion of a safety culture and the involvement of all stakeholders. Therefore, it is important to define a benchmark framework that, based on the best evidence, integrates, articulates and guides the action of all stakeholders to reduce the occurrence of harm [11].

In Portugal, PS is an unavoidable subject, evident in several official documents, which reveal a concern to align national strategies with international guidelines. It is noteworthy that one of the National Health Plan’s strategic axes foresees specific measures in the scope of safety, namely the implementation of a National Plan for the Safety of Patients 2015–2020 [12], which aims to support managers and clinicians in managing the risks associated with the provision of care [13].

Despite this growing concern, scientific production on this matter in the national context is still scarce. With regard to the epidemiological dimension of the problem, we highlight a study that revealed an AE incidence rate of 12.5% in hospitalisation incidents, of which 39.9% would be preventable. It should be noted that one of the limitations of this study is the non-inclusion of the paediatric population [14].

In short, the challenges in Portugal relate to (1) the deeper epidemiological knowledge of AE; (2) the uniformity of the notification systems and the articulation of the information systems, in order to allow a consistent comparison of data; (3) the paradigm shift in terms of security is seen as an investment and not an expense; (4) investing in research and strengthening partnerships between clinical and academic contexts; (5) and the introduction of the topic in initial and advanced training [15].

### Paediatric safety

Access to high quality health care is an essential human right [16]. The right to safety has been reported in several significant documents in the field of Paediatrics, such as the Convention on the Rights of the Child [17] and the Notes to the Charter of the Hospitalised Child [18]. Despite this fundamental right, several studies point to a high incidence rate of AE in Paediatrics, despite the limited and variable data in different hospital contexts.

It is estimated that approximately 70,000 children per year suffer some form of harm from health-related events [19]. It is described that they are more prone to the occurrence of an AE and those who experienced it had a longer hospital stay, higher mortality rate and resulted in a greater institutional burden, which accurately reflects the human and economic impact of this problem [20–23].

Children have specific risk factors that make them more susceptible to harm and, according to several authors, contribute to about half of the events. These factors can help us understand some causes of AE and refer to (1) physical characteristics, which increase the events with therapy, ventilation and vascular accesses; (2) developmental characteristics, which are related to events associated with communication, patient identification and monitoring; and (3) dependency level, which influences their participation in decision making and care [21, 23–25].

### Safety in paediatric intensive care

The incidence of events varies according to the place where they occur, with factors underlying the patient themselves and with the type of care needed. Fragata [1] states that paediatric patients are more likely to experience an AE with a high severity potential and that, in intensive care, it is estimated that each suffers from one to two AE per day, which makes the Paediatric Intensive Care Units [PICU] a potentially lethal context. PICU is defined as a service for hospitalisation of patients aged between 1 month and 18 years of age, with serious and potentially reversible diseases, which can benefit from a more detailed observation than that which is usually available in general wards [26].

AE are a common problem in this context and, at international level, their incidence and typology is widely variable [27], which may be associated to the level of PICU, the methodology used, the type of incident involved and the awareness of professionals about the notification. As for the methodology, prospective approaches such as direct observation and surveillance may be used, or retrospective approaches like the review of documents or clinical processes and the analysis of incidents reported voluntarily in the notification systems [28]. As there is no universal measurement instrument for PS, it is necessary to know the advantages and limitations of the chosen methods. The assessment of different types of harm requires a multiplicity of measures, and knowledge about their specificity, validity, reliability and applicability to different contexts is essential [28–31].

One of the most widely used methodologies for measuring AE is document review using the Trigger Tool (TT) [28, 32–34]. This methodology consists of a retrospective review of clinical processes, in order to identify clues/triggers that suggest the occurrence of a certain damage resulting from an AE. This review is carried out by at least three reviewers trained in this methodology and takes place in two essential phases: (1) two primary reviewers, usually nurses, with vast knowledge and experience in the care context, make an independent review of the processes in order to detect the presence of the triggers, and (2) a doctor authenticates the consensus of the primary reviewers and determines the severity level of the AE. This doctor, despite not carrying out the review of the process, must be available to answer questions that arise in the first phase [33, 35].

One of the main drivers of the use of TT was the IHI, which developed several tools for different clinical contexts. Specifically in Paediatrics, some tools have been developed in this line of methodology, of which the British and Swedish versions stands out [32, 36]. In the context of PICU, a systematic literature review [32] was identified: the PICU trigger list (pilot study) [37], the PICU Trigger Tool (which assembled and validated other TT triggers) [38], and the PICU Trigger Tool (developed by Child Health Corporation of America).

A study carried out at a PICU that compares the TT methodology with the analysis of incidents reported voluntarily concluded that the first is simple, efficient and robust and allows the detection of a greater number of AE. However, it does not detect near miss, diagnostic and omission errors and many types of medication errors, and it does not provide any feedback on the contributing factors [39]. Bearing in mind that voluntary notification allows the overcoming of these limitations, this methodology, despite identifying only 10–20% of AE [40], should be used complementarily.

With the multicentre study carried out by Agarwal et al as a reference [38] in 15 US PICU and using a TT, the incidence was 2 AE per patient and significantly higher rates in patients who died. Another study that covered 23 PICU, also in the USA, concluded that two thirds of the incidents caused harm and that child-related factors were the strongest predictor of that damage. Despite the variability of studies, the most common AE were those related to medication, ventilation, catheters, equipment, infections associated with healthcare, poor regulation of alarms, lack of training and professional training and ineffective communication [1, 23, 27, 41–44]. Despite medication errors being the most frequently mentioned, it was those associated with ventilation and vascular access that represented a higher level of harm [23].

This high incidence is due to the clinical complexity of the patients, the pressure associated with urgent situations, the length of stay, the invasive nature of the procedures, the therapeutic intensity and the use of complex equipment [1, 45]. The costs associated with this problem must be properly analysed, so that the promotion of safety is seen as an investment and not an expense. As an example, it is estimated that the costs directly attributed to infections in the central vascular accesses are around $ 55,000, accidental extubations to $ 101,000 and the most serious AE to $ 440,000 [45].

In order to improve the safety of the care provided in this context, after understanding the causes mentioned above, it is necessary to identify the most effective solutions, with care bundles, checklists, double check systems, structured shift passages and point of care, rapid response systems and information and communication technologies being some of the resources suggested in the literature [27, 45–48]. In order to assess the impact, specific quality indicators can be used to evaluate the outcomes, such as mortality rate, rate of unplanned readmissions, the length of stay adjusted to the clinical severity and the rate of injuries, therapeutic errors, unscheduled extubations, incidents associated with vascular access, infections associated with healthcare and equipment-induced damage [1, 21, 49]. All of this research data should be disseminated by managers and direct care providers and solutions should be adequate for local contexts, to foster commitment to a practice based on levels of evidence [50].

In short, several incident measurement instruments are scattered across the scientific literature, so it is crucial that there is a synthesis that facilitates access to information by the various stakeholders in this area and allows them to choose, in a more judicious way, the instrument that best suits a particular population and context.

### Objectives and research questions

The objective of this Scoping Review [ScR] is to identify and map in the scientific literature the instruments for measuring incidents related to patient safety applicable in the context of paediatric intensive care.

The defined review questions are:

1. What instruments are used to measure incidents related to patient safety in the context of paediatric intensive care?
2. What instruments can measure near miss and harmless events related to patient safety in the context of paediatric intensive care? What instruments allow the specific measurement of adverse events related to patient safety in the context of paediatric intensive care? Can these measurement tools be used for all types of incidents and what are their mains characteristics?
3. Is there evidence of the effectiveness of the application of these methods to improve patient safety?

## Methods

This study protocol has been registered within the Open Science Framework (registration number: osf.io/b5m7j) and is being reported in accordance with the reporting guidance provided in the Preferred Reporting Items for Systematic Reviews and Meta-Analyses Protocols (PRISMA-P) statement [51, 52] (see checklist in Additional file 1). The planned review will be reported according to the PRISMA Extension for scoping review (PRISMA-ScR) [53] (see checklist in Additional file 2). This scoping review protocol will be conducted by using Joanna Briggs Institute (JBI) guidelines for scoping reviews, ensuring systematic and repeatable work [54] and will follow the five stages included when conducting a scoping review as outlined by Arksey & O’Malley [55].

### Eligibility criteria

#### Participants

This ScR will include all studies, which refer to instruments for measuring incidents related to patient safety, whose participants are children and young people hospitalised in the context of paediatric intensive care.

#### Concept

The phenomenon of interest for a ScR is related to the instruments for measuring incidents related to patient safety in the context of paediatric intensive care. These instruments can be incident reporting systems, trigger tools or morbidity and mortality conferences, being able to measure the different types of incidents, as adverse events or near misses.

#### Context

The defined context for a ScR is all paediatric intensive care units, regardless of their level of care activity, their polyvalent or specific typology, the type of hospital unit they belong to or their geographical location.

#### Study designs

This ScR will cover all scientific articles that include studies on the subject, of a quantitative, qualitative or mixed nature and published and unpublished literature reviews (grey literature). Analytical documents (reports from reputable organisations, expert opinions or comments) that report or analyse aspects considered relevant to the subject in question will also be considered.

The studies may result from a single or multidisciplinary view (nursing, medicine, psychology, etc.). From a linguistic point of view, documents will be limited to Portuguese, English and Spanish. Timewise, documents published from 2015 onwards—the previous 5 years from the beginning of the review—will be included, since the research syntheses conducted within the last 5 to 10 years will yield original research conducted previously, according to the JBI [56]. However, after this first phase of research, studies and documents prior to 2015, but which contain measurement instruments relevant to the context in question, may be considered.

#### Information sources

To identify documents potentially relevant to the ScR, and in order to increase the sensitivity of the research, two types of information sources will be used:

1. Electronic databases - MEDLINE Complete, CINHAL Complete, MedicLatina and Cochrane Library (Cochrane Database of Systematic Reviews) via EBSCO; Science Direct and Scopus for Elsevier publications; Joanna Briggs Institute EBP Database via OVID; BioMed Central; and Scientific Electronic Library Online (SciELO Portugal and Brazil);
2. Other documents, namely documents included in the Open Access Scientific Repository of Portugal; documents issued by the main organisations focused on patient safety (WHO, AHRQ, IHI, National Patient Safety Agency, Joint Commission International, National Quality Forum and PROQUALIS), some of which are included in the “Gray Matters: a practical tool for searching health -related grey literature ”that the Canadian Agency for Drugs and Technologies in Health outlined for approaching grey literature research [57]. These documents may include reports, guidelines or opinion articles.

#### Search strategy

The research strategy defined for this ScR involves three distinct stages:

1. Initial research carried out in a selection of relevant databases (MEDLINE and CINAHL), to analyse and select the main terms used (indexed and in natural language) in the titles and abstracts of articles related to patient safety in a paediatric intensive care context (;
2. Research in each of the previously-mentioned sources of information, using the terms selected in the previous step and adapting them to each one specifically. After reading the titles and abstracts, two reviewers will select and read in full the documents that meet the eligibility criteria;
3. Analysis of the reference list of the selected documents, to identify additional bibliography.

The design of the search strategy carried out at MEDLINE with the respective selected terms and defined limits is found in Additional file 3.

The design of the research strategy and the selection of documents in the second stage will be carried out by both reviewers, independently and using previously established screening questions.

### Data charting process

#### Data management

The selected documents will be uploaded to the Covidence web tool, which, in partnership with Cochrane, allows the optimisation of the systematic review process and facilitates collaboration between reviewers. For the management of references, the Mendeley application will be used.

#### Data selection process

Two reviewers will independently carry out the four stages of data selection: identification, selection, eligibility and inclusion. In case of doubt, a debate will take place and, if necessary, a third reviewer will be contacted.

#### Data collection process

To gather the data collected in each of the selected documents, a chart, which will group the most relevant information and answer the defined review questions, as well as the characteristics of the studies/documents, was created and validated by the two reviewers. This selection is an interactive process and as such the chart will be continuously adjusted as the extraction proceeds. If a significant number of studies/documents are selected, a pilot test will be carried out to check the adequacy of the chart.

#### Critical appraisal of individual sources of evidence

Quality assessment will not be carried out, as it is not a mandatory criterion for a ScR (53) and its aim is to encompass as much information about available measuring instruments.

#### Synthesis of results

The results of the research strategy will be presented in the PRISMA flowchart. The extracted data, after being organised in the extraction chart (see Additional file 4), will be mapped in a descriptive and logical way, considering the defined review questions. Thus, a summary is sought that identifies the instruments for measuring incidents related to patient safety in the context of paediatric intensive care and that summarises their characteristics, specificities and limitations. These results will be presented in a narrative way and, additionally, the studies and documents characteristics will be presented in a tabular format.

## Discussion

The vulnerability of the paediatric population, combined with the potentially dangerous context of intensive care, makes PICU services of particular complexity in matters of safety, where there is a greater likelihood of incidents with serious consequences. To identify high-risk areas and define priorities it is necessary to resort to a multiplicity of measurement instruments.

As there is no universal measurement instrument for PS, it is necessary to know the advantages and limitations of the chosen methods. The assessment of different types of harm requires a multiplicity of measures and the knowledge about their specificity, validity, reliability and applicability to different settings is essential. Therefore, it is crucial to map instruments that can identify incidents in the paediatric population but are also accurate for more specific events related to the ICU setting.

The mapping of such tools in this protocol will allow to summarise the most widely used instruments, to know their specificities and to guide researchers to use the most appropriate measurement tools for their context, specifically in paediatric intensive care. Moreover, the choice for an instrument must consider suitable levels of evidence and degrees of recommendation [58].

## Limitations

Limitations of our findings are anticipated due to heterogeneity in the instruments studied and differences in the context in which they are applied. Plus, limited access to information sources and linguistic and time limits may exclude some relevant sources, but it is intended that additional research can overcome this limitation.

## Dissemination

The results of the review will be disseminated through publication in a peer-reviewed journal, interaction with potential knowledge users as PICU professionals and presentation in conferences or seminars.

## Amendments

Any amendments to this protocol will be documented with reference to saved searches and analysis.

## Supplementary Information

**Additional file 1.** PRISMA-P 2015 Checklist.

**Additional file 2.** Preferred Reporting Items for Systematic reviews and Meta-Analyses extension for Scoping Reviews (PRISMA-ScR) Checklist.

**Additional file 3.** Research Strategy Design (MEDLINE).

**Additional file 4.** Data Extraction Table.

## Data Availability

Not applicable.

## Abbreviations

PS: Patient safety
IOM: Institute of Medicine
WHO: World Health Organization
AE: Adverse events
IHI: Institute for Healthcare Improvement
USA: United States of America
PICU: Paediatric intensive care units
TT: Trigger tool
ScR: Scoping Review
