# Supplementary material for: Instruments for measuring incidents related to patient safety in the context of paediatric intensive care—protocol for a scoping review
Source: Syst Rev. 2022 Jan 25;11:17. doi: 10.1186/s13643-022-01888-6 (PMC8790838; doi:10.1186/s13643-022-01888-6)
Supplement: Supplementary file 3 — Additional file 3. Research Strategy Design (MEDLINE). [file 13643_2022_1888_MOESM3_ESM.docx]

# Additional file 3

##

## Research Strategy Design (MEDLINE)

|  | Terms r / c  Participants | Terms r / c  Concept | Terms r / c  Context |
| --- | --- | --- | --- |
| Indexed (MeSH 2018) | **S1** MH "Paediatrics" | **S4** MH "Patient Safety"  **S5** MH "Medical Errors"  **S6** MH "Patient Harm" | **S12** MH "Intensive Care Units, Pediatric" |
| Natural Language | **S2** pediatric **S3** paediatric | **S7** patient safety  **S8** medical errors  **S9** patient harm   **S10** adverse event **S11** trigger tool | **S14** paediatric intensive care  **S15** paediatric critical care |

**Search Expression:**

(S1 OR S2 OR S3) AND (S4 OR S5 OR S6 OR S7 OR S8 OR S9 OR S10 OR S11) AND (S12 OR S13 OR S14 OR S15)

**Limiters** - Publication Date: 20150101-20201231

**Restrict by Language:** English

**Restrict by Language:** English

**Restrict by Language:** Spanish
