# Supplementary material for: Instruments for measuring incidents related to patient safety in the context of paediatric intensive care—protocol for a scoping review
Source: Syst Rev. 2022 Jan 25;11:17. doi: 10.1186/s13643-022-01888-6 (PMC8790838; doi:10.1186/s13643-022-01888-6)
Supplement: Supplementary file 4 — Additional file 4. Data Extraction Table. [file 13643_2022_1888_MOESM4_ESM.docx]

# Additional file 4

## Data Extraction Table

| Study ID | Characteristics | | | Eligibility Criteria | | | Results | | | | Limitations | Suggestions |
| --- | --- | --- | --- | --- | --- | --- | --- | --- | --- | --- | --- | --- |
| Title  Authors  Year of Publication  Country of Origin | Aim/  Purpose | Methods  (Study design) | Quality and ethical considerations | Participants | Concept  (Instruments) | Context | Outcome  measures | Incident type  and category | Harm | Costs |  |  |
|  |  |  |  |  |  |  |  |  |  |  |  |  |
|  |  |  |  |  |  |  |  |  |  |  |  |  |
|  |  |  |  |  |  |  |  |  |  |  |  |  |
|  |  |  |  |  |  |  |  |  |  |  |  |  |
